# Smoking cessation and survival in lung, upper aero-digestive tract and bladder cancer: cohort study

**DOI:** 10.1038/bjc.2017.179

**Published:** 2017-09-12

**Authors:** C Koshiaris, P Aveyard, J Oke, R Ryan, L Szatkowski, R Stevens, A Farley

**Affiliations:** 1 Nuffield Department of Primary Care Health Sciences, UK Centre for Tobacco and Alcohol Studies, University of Oxford, New Radcliffe House, Radcliffe Observatory Quarter, Woodstock Road, Oxford OX2 6GG, UK; 2 Institute of Applied Health Research, University of Birmingham, Edgbaston, Birmingham B15 2TT, UK; 3 Division of Epidemiology and Public Health, University of Nottingham, Clinical Sciences Building, Nottingham City Hospital, Nottingham NG5 1PB, UK

**Keywords:** smoking, smoking cessation, cancer, primary care

## Abstract

**Background:**

The aim was to examine the association between smoking cessation and prognosis in smoking-related cancer as it is unclear that cessation reduces mortality.

**Methods:**

In this retrospective cohort study from 1999 to 2013, we assessed the association between cessation during the first year after diagnosis and all-cause and cancer-specific mortality.

**Results:**

Of 2882 lung, 757 upper aero-digestive tract (UAT) and 1733 bladder cancer patients 27%, 29% and 21% of lung, UAT and bladder cancer patients quit smoking. In lung cancer patients that quit, all-cause mortality was significantly lower (HR: 0.82 (0.74–0.92), while cancer-specific mortality (HR: 0.89 (0.76–1.04) and death due to index cancer (HR: 0.90 (0.77–1.05) were non-significantly lower. In UAT cancer, all-cause mortality (HR: 0.81 (0.58–1.14), cancer-specific mortality (HR: 0.84 (0.48–1.45), and death due to index cancer (HR: 0.75 (0.42–1.34) were non-significantly lower. There was no evidence of an association between quitting and mortality in bladder cancer. The HRs were 1.02 (0.81–1.30) for all-cause, 1.23 (0.81–1.86) for cancer specific, and 1.25 (0.71–2.20) for death due to index cancer. These showed a non-significantly lower risk in sensitivity analyses.

**Conclusions:**

People with lung and possibly UAT cancer who quit smoking have a lower risk of mortality than people who continue smoking.

Around a fifth of all cancers worldwide are caused by smoking (Ezzati *et al*, 2005), and smoking-related tumours commonly develop in the lung, upper aero-digestive tract (UAT) and bladder (Thun *et al*, 2009) (US Surgeon General, 2014b). Although 5-year survival rates for lung cancer are low, as most patients present in late stages (Verdecchia *et al*, 2007; Walters *et al*, 2013), around 70% of patients who are treated curatively survive for 5 years (UK. CR, 2016). The proportion of people with curatively treated cancer is likely to increase with the advent of computerised tomography-based lung cancer screening. In upper aerodigetsive tract (UAT) and bladder cancer, the European mean age-standardised 5-year survival rates are ∼40 and 70%, respectively (De Angelis *et al*, 2014). Thus, there is a large group of people with smoking-related cancer who may benefit from additional interventions to improve prognosis.

There is some evidence that quitting smoking after diagnosis of smoking-related cancers may be associated with improved prognosis, particularly in patients who have been diagnosed in early stages (Aveyard *et al*, 2002; Parsons *et al*, 2010; US Surgeon General, 2014b). However, many of these studies have methodological limitations such as a small sample size and unclear definitions of smoking status (Gritz *et al*, 2014). In addition, most studies compare the prognosis in patients who are smoking at diagnosis to those who have quit some time before or who have never smoked. Few studies have focused on the prognostic benefit of quitting at or soon after diagnosis (Land, 2012; Balough *et al*, 2014; US Surgeon General, 2014b).

Many patients with smoking-related cancers are still smoking at diagnosis (Cooley *et al*, 2009; Park *et al*, 2012; Warren *et al*, 2013a) and support to quit is not routinely offered as part of cancer care (Murray *et al*, 2012; Warren *et al*, 2013b). If quitting soon after diagnosis improved prognosis, this would increase the clinical imperative to offer patients smoking cessation interventions to improve their chances of survival. Unlike many treatments for cancer, smoking cessation treatment is safe and has mild adverse effects. In order to investigate if quitting after diagnosis has prognostic benefits, we estimated the association between quitting and all-cause mortality in lung, UAT and bladder cancer patients using a large data set of the routinely-collected primary care data. Cessation reduces mortality from cardiorespiratory disease and thus cessation would be expected to reduce mortality from this cause. We therefore investigated whether some of the benefit could be due to prevention of cancer progression by examining the association between quitting and death due to cancer.

## Materials and methods

We conducted a retrospective cohort study using routinely-collected UK primary care records from the Clinical Practice Research Datalink (CPRD) (www.cprd.com). The protocol was approved by the Independent Scientific Advisory Committee (ISAC) for MHRA database research (reference no: 14_105), and was made available during the peer review process. Patients with a first diagnosis of lung, UAT and bladder cancers between 1999 and 2013 who smoked at diagnosis, survived for one year, had been registered with a practice for at least one year and had at least one year of follow-up data were included in the cohort. UAT cancers mainly included cancers that occur in the mouth and throat, and full definitions, and the Read codes used for each cancer are provided in Appendix 1 (HSCIC, 2014). We restricted the analysis to patients that survived for at least 1 year to limit confounding by stage of cancer at diagnosis. Stage was not recorded in most cases. People with advanced cancer-treated palliatively may be less likely to stop smoking and are more likely to die, thus confounding the association between smoking cessation and mortality. As many such patients die within a year, restricting the analysis in this way limited confounding. Patients were followed-up until the end of 2013 or death, or were censored if they moved practice. In 2013, the CPRD database contained records from 4.4 million live patients in 674 practices, which represented 6.9% of the UK population (Herrett *et al*, 2015). The first version of the protocol was amended to exclude people with thyroid cancer because this is not a smoking-related head and neck cancer.

Patients were defined as smokers at diagnosis if they were recorded as smoking on the last occasion smoking status was updated within the 3 years prior to diagnosis. People were classified as having stopped smoking or continued smoking during the first year of follow-up if their last record during that period recorded either state. Some people did not have their smoking status updated after diagnosis, and we conducted sensitivity analyses to examine the impact of this on the findings.

Survival time was calculated as the time from the end of the first year after diagnosis until outcome occurrence (all-cause mortality, cancer-specific mortality or death due to index cancer). The fact of death was taken from the CPRD record and also from the UK national system of recording death provided by the Office for National Statistics (ONS) and this was used for all-cause mortality analyses. The CPRD database does not record cause of death but ONS does. Thus, for those patients in practices where the ONS death data could be linked, we examined cancer-specific mortality and death due to index cancer. To do so, the ONS data were linked to each person’s CPRD data. In the protocol, we planned to investigate development of a recurrence or development of a second primary tumour but were unable to do this as these outcomes were poorly recorded.

Survival curves were generated with the Kaplan–Meier plot method for each outcome. We used Cox proportional hazard regression models to compare differences in survival between people who stopped and people who continued smoking in each cancer group. The proportional hazards assumption was examined by the use of log–log plots and Schoenfeld residuals. The results are presented as hazard ratios with 95% confidence intervals, and we use the term non-significant as shorthand to mean that the 95% confidence intervals included unity. In the main analyses, we adjusted for age, gender, socio-economic status (SES), comorbidity (asthma, chronic kidney disease (CKD), chronic obstructive pulmonary disease (COPD), diabetes, hypertension, peripheral arterial disease (PAD), stroke, psychoses), treatment and alcohol consumption. Patients whose smoking status after diagnosis was missing, were included as an additional exposure category. Due to missing data on smoking status after diagnosis, alcohol status and SES we used multiple imputation (MI) models to impute these data. We used the mi command in STATA 14 with 20 imputed datasets, including the baseline and clinical characteristics, outcome and the Nelson–Aalen estimator of the cumulative hazard function (White and Royston, 2009).

Sensitivity analyses were conducted to investigate the robustness of MI models. Sensitivity analyses comprised unadjusted analysis, full case analysis and full case analysis, where patients that did not have their smoking status updated within the year following diagnosis were classified as continued smokers. We performed two additional sensitivity analyses but do not show the data as the results of these analyses were very similar to the main results. First, we excluded covariates with missing data (alcohol status and SES). Second, in response to a referee, we adjusted for year of diagnosis, which made no material difference, and then added a multiplicative interaction term between smoking status and year of diagnosis, and this was not significant in any analysis.

## Results

**Figure 1:**
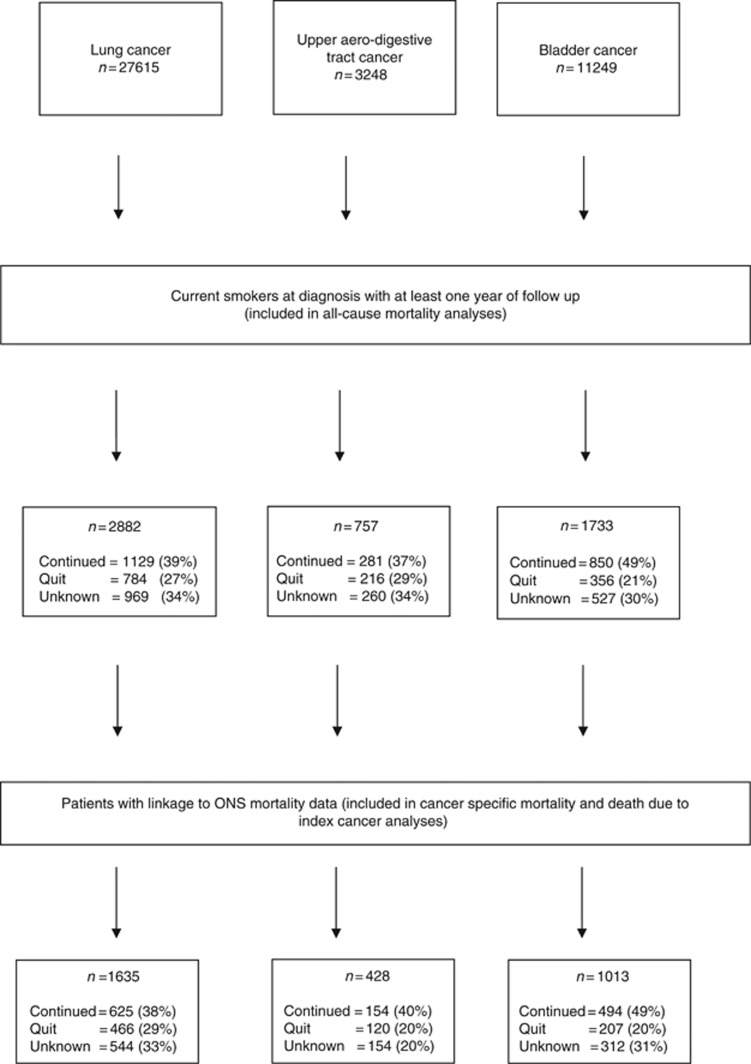
**Flow of participants through the study.**

During the study period, there were a total of 42 112 incident cancer cases (lung *n*=27 615, UAT *n*=3248, bladder *n*=11 249). Information regarding smoking status at diagnosis was available for 81% of these cases (lung 83%, UAT 78%, bladder 79%), and we assumed that patients with missing status were either never smokers, or long-term ex-smokers, consistent with the rules GPs for updating smoking status (Marston *et al*, 2014). On the basis of this assumption, 36%, 35% and 20% of lung, UAT and bladder cancer patients were smoking at diagnosis. We included in our analyses patients who smoked at diagnosis and who had survived for at least 1 year. This included 2882 people with lung cancer (27% quit, 39% continued, 34% unknown), 757 people with UAT cancer (29% quit, 37% continued, 34% unknown) and 1733 people with bladder cancer (21% quit, 49% continued, 30% unknown). As not every practice in the CPRD database is linked to ONS for the cause of death analyses, the cohorts for the analyses of cancer-specific mortality and death due to index cancer were smaller (lung cancer *n*=1635; UAT cancer *n*=428; bladder cancer *n*=1013) (Figure 1).

The baseline demographic characteristics were similar across smoking exposure groups (quit during first year, continued during first year, no smoking update during first year) for each cancer group. There were some imbalances in the presence of comorbidities across exposure groups for each cancer group (Table 1). A higher proportion of patients who quit smoking received surgery. However, these baseline characteristics were controlled for in the analyses.

### Association between smoking status after diagnosis and all-cause mortality

**Figure 2:**
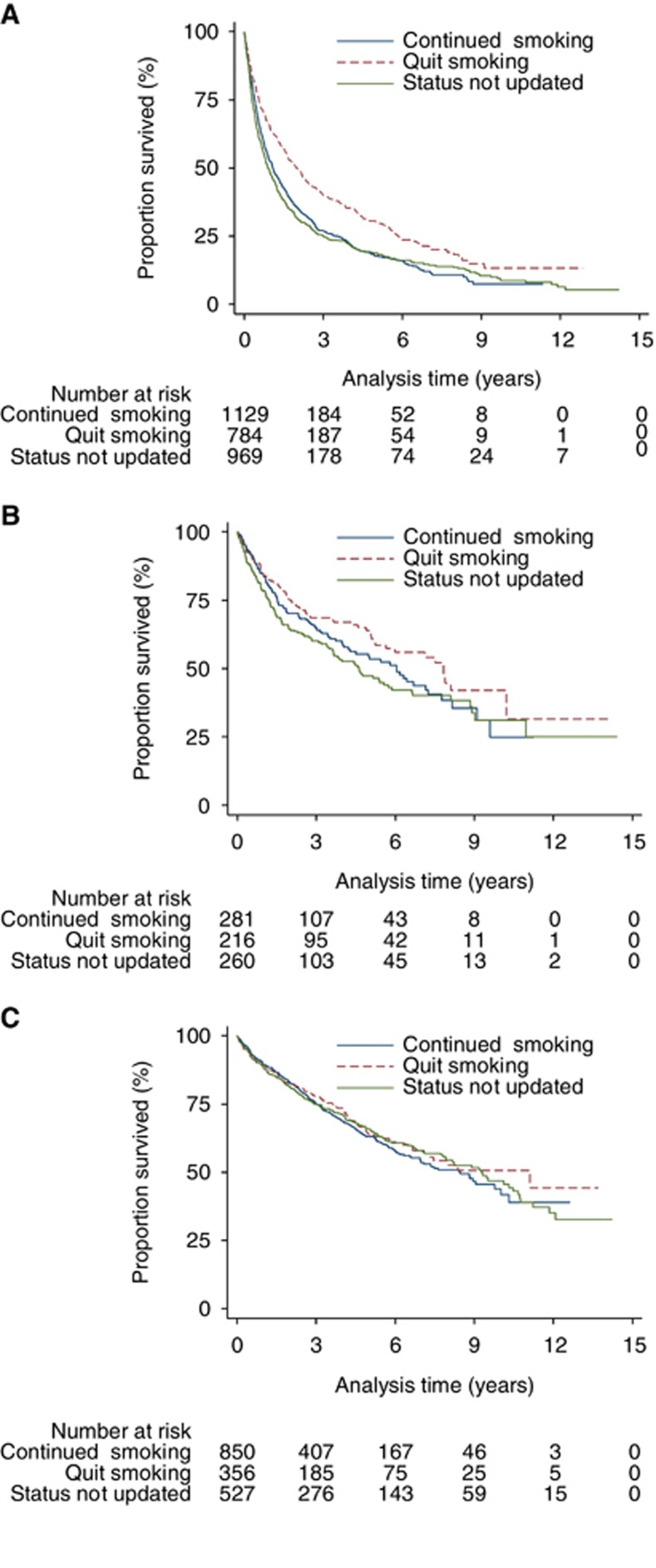
**Unadjusted risk of all-cause mortality in patients who quit smoking compared with those who continued to smoke after diagnosis.** (**A**) Lung cancer; (**B**) Upper aero-digestive tract cancer; (**C**) Bladder cancer.

Lung cancer patients who quit smoking had a significantly lower risk of all-cause mortality compared with patients who continued to smoke (unadjusted HR: 0.71 (95% CI: 0.63–0.79), adjusted HR: 0.82 (95% CI: 0.74–0.92)) (Table 2). Median survival (from 1 year after diagnosis) for patients who continued smoking was 1.08 years (95%CI: 0.94–1.24), and was 1.97 years (1.68–2.26) for patients who quit. In UAT, quitting smoking was associated with a non-significantly lower risk of mortality, which was unchanged after adjustment (unadjusted HR: 0.80 (0.60–1.08), adjusted HRL 0.81 (0.58–1.14)). There was no evidence of an association between quitting and mortality in patients with bladder cancer (unadjusted HR: 0.91 (95% CI: 0.73–1.14), adjusted HR: 1.02 (0.81–1.30)) (Figure 2). The sensitivity analysis with different assumptions produced very similar results to these for all cancers (Table 2).

### Association between smoking status after diagnosis and cancer-specific mortality

**Figure 3:**
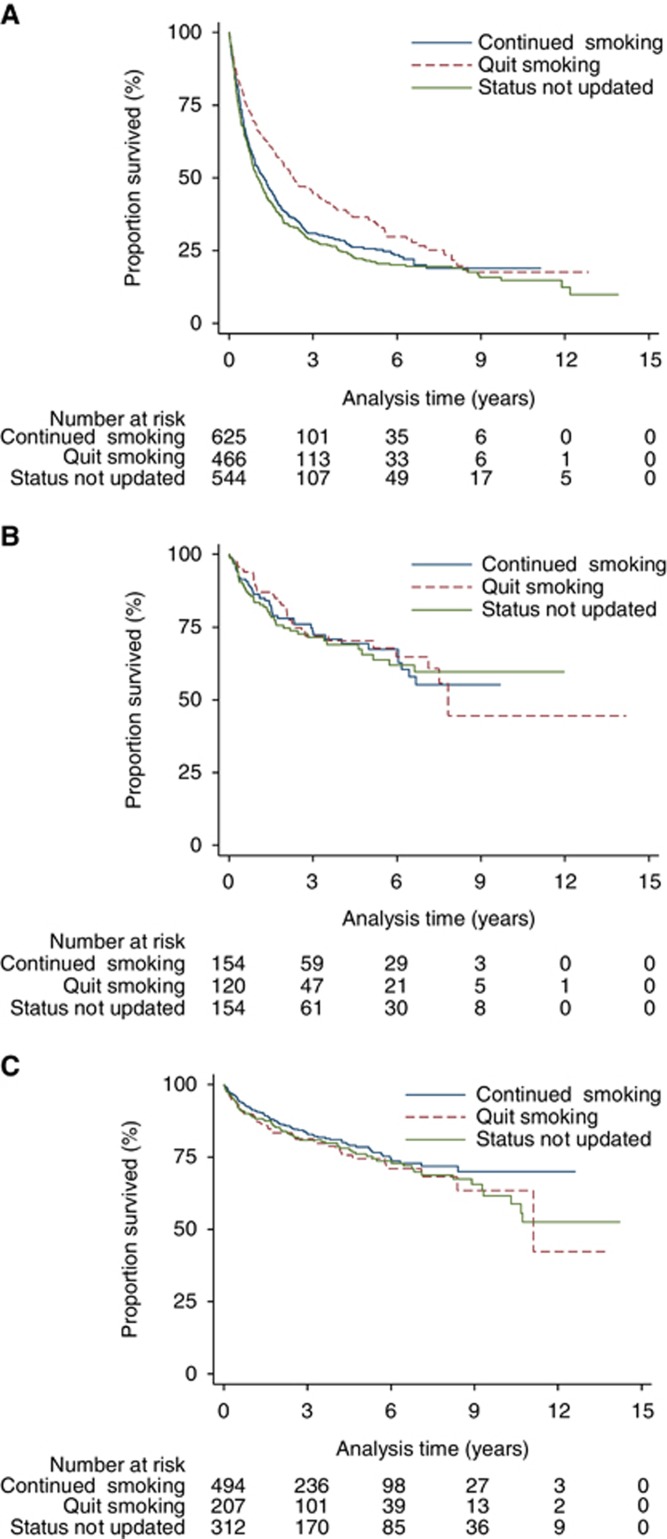
**Unadjusted risk of cancer-specific mortality in patients who quit smoking compared with those who continued to smoke after diagnosis.** (**A**) Lung cancer; (**B**) Upper aero-digestive tract cancer; (**C**) Bladder cancer.

Of all the people diagnosed with lung cancer, 86% died of lung cancer. Quitting smoking after a diagnosis of lung cancer was associated with lower cancer-specific mortality (unadjusted HR: 0.73 (0.62–0.85)), but this became non-significant after adjustment (HR: 0.89 (0.76–1.04)) (Table 2). Less than two thirds of the deaths after UAT and bladder cancer were due to cancer (65 and 59%, respectively). Quitting smoking after diagnosis of either cancer was not significantly associated with reduced cancer-specific mortality (Table 2, Figure 3). Sensitivity analysis again showed very similar results.

### Association between smoking status after diagnosis and death due to index cancer

**Figure 4:**
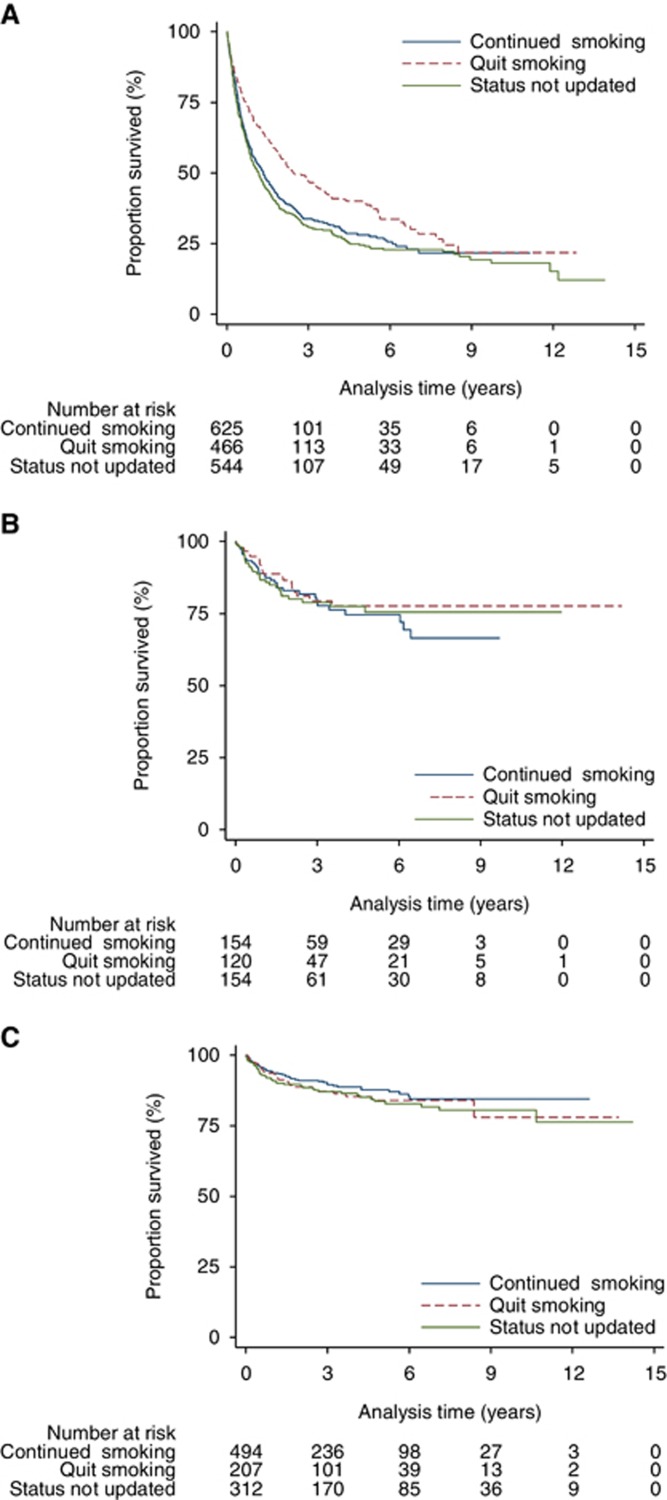
**Unadjusted risk of death due to index cancer in patients who quit smoking compared with those who continued to smoke after diagnosis.** (**A**) Lung cancer; (**B**) Upper aero-digestive tract cancer; (**C**) Bladder cancer.

There was no significant association between quitting smoking and death due to index cancer in lung (unadjusted HR: 0.72 (0.61–0.85), adjusted HR: 0.90 (0.77–1.05, UAT (unadjusted HR: 0.78 (0.45–1.37), adjusted HR: 0.75 (0.42–1.34) or bladder cancer patients (unadjusted HR: 1.24 (0.77–1.99), adjusted HR: 1.25 (0.71–2.20)) (Table 2, Figure 4). Sensitivity analysis produced similar results for lung and UAT cancers but there was a modest difference between the multiple imputation and the full case models for bladder cancer. However, neither set of models suggested a significant increase or decrease in risk for people who stopped smoking.

## Discussion

A third of patients with lung and UAT cancer, and a fifth of those with bladder cancer smoked at diagnosis, and the majority continued after their diagnosis. In patients who survived for at least 1 year, smoking cessation during that first year was associated with a lower risk of all-cause mortality in lung cancer and the evidence was suggestive of a lower risk in UAT cancer. There was inconclusive evidence that quitting was associated with survival in bladder cancer. There was inconclusive evidence that smoking cessation was associated with lower mortality in each cancer type.

This study included many more people than previous studies investigating the impact of smoking cessation on survival, therefore producing more precise estimates of association. However, there were fewer people with data available on cause of death. This meant that the estimates for cancer-specific outcomes were less precise and it was not possible to be sure whether the apparent survival benefit arose from reduced deaths from cardiac and respiratory causes or from reduced deaths due to cancer in UAT cancer because the confidence intervals for cancer-specific deaths were wider. However, for lung cancer, it appears clearer that quitting smoking is associated with a lower cancer-specific mortality, mainly because the large majority of deaths were due to lung cancer, although the adjusted estimates of cancer-specific mortality (based on a subsample) were not themselves significant.

This study has some limitations. Firstly, smoking status was taken from the medical records and these may not accurately record true smoking status. Doctors did not record whether around a third of people continued or stopped smoking, though sensitivity analysis suggest the results were insensitive to this. Some people who were recorded as having stopped smoking may have subsequently relapsed, while some recorded as continuing may subsequently stopped. This mixing of exposure assignment is likely to underestimate the strength of association (Sorahan and Gilthorpe, 1994; Wacholder *et al*, 1995), but it creates uncertainty. Although we controlled for a range of confounders, we were unable to adjust for all possible confounders. In particular, the data on stage at diagnosis is not recorded in UK primary care notes so this could not be controlled for in the analysis. There is no biologically plausible reason why those who quit smoking after diagnosis would present with more favourable stage than those who continued to smoke. However, it is plausible that presenting with more advanced cancer undermines motivation to stop smoking and that this would confound the association, producing the associations in the direction we observed. In addition, primary care records have limited information on the treatment received and often this was not recorded. Consequently, it was not possible to reliably differentiate those treated curatively from those treated palliatively. We confined the analysis to people who survived at least a year to try to limit this confounding, but this may not have been entirely successful. In addition, SES and alcohol consumption were not well recorded, and more than 20% of people had missing data for these variables so we used multiple imputation models. These models produced broadly similar results to the models that did not control for these variables.

### Interpretation and context with previous literature

Our findings relating to all-cause mortality are consistent with other published literature (Browman *et al*, 1993; Aveyard *et al*, 2002; Clark *et al*, 2006; Mayne *et al*, 2009; Parsons *et al*, 2010) We have previously reported a systematic review of studies in lung cancer patients that found that, after adjustment for key prognostic factors, quitting was associated with reduced mortality, with a HR of 0.34 (95%CI: 0.13–0.87) for non-small cell cancer and 0.54 (95%CI: 0.39–0.75) for small cell lung cancer (Parsons *et al*, 2010). Both these estimates were derived from only one study each, with around 200 participants. Our study produced more modest estimates of the benefits of quitting in lung cancer, but the point estimates are within these confidence intervals. Similarly, studies comparing mortality between people who quit smoking after diagnosis of head and neck cancer and people who continue to smoke have found lower mortality risk in those who quit. For example, Mayne *et al*, 2009 reported a significantly lower risk of mortality (HR: 0.30 95%CI: 0.16–0.57) (Mayne *et al*, 2009) and Browman *et al*, 1993 reported improved two-year survival (66% *vs* 39%, *P*=0.005). Studies in patients with bladder cancer have compared rates of all-cause mortality in people who smoke at diagnosis to those who have never smoked, or to those who have quit some time before diagnosis, but not to people who quit after diagnosis and therefore there is no evidence from previous studies to compare our results with (Aveyard *et al*, 2002; Van Osch *et al*, 2016). In our analysis, we adjusted for common comorbidities, and smoking cessation was still associated with a risk of death due from any cause in lung cancer. There is strong evidence elsewhere that quitting smoking reduces the risk of heart disease and respiratory death (Critchley and Capewell, 2003), including from a clinical trial (Anthonisen *et al*, 2002), and therefore we conclude that the association between quitting and reduced all-cause mortality in lung cancer is causal.

There is evidence that continued smoking increases risk of a second primary cancer and recurrence (US Surgeon General, 2014a), but the evidence that quitting at the time of diagnosis reduces this risk is relatively weak. For example, in our review of lung cancer patients, both non-small cell and small cell lung cancer patients who quit smoking had a lower risk of recurrence (NSCLC HR: 0.54 (0.29–0.99); SCLC HR: 0.79 (0.67–0.94) and small cell lung cancer patients had a lower risk of second primary tumour although confidence intervals were wide (HR 0.23 (0.05–0.92) (Parsons *et al*, 2010). Each of these estimates originate from one study only which include a small numbers of patients (Kawahara *et al*, 1998; Videtic *et al*, 2003; Nia *et al*, 2005). Do *et al* found that in patients with early stage head and neck squamous cell carcinoma, continued smokers and quitters were both more likely to develop second primary tumours than never smokers. However, when indirectly comparing continuing smokers and quitters (Bucher *et al*, 1997)/ there was no evidence of a difference in risk (HR 0.94 (95%CI 0.34–2.60). Leon *et al* conducted a matched case control study in patients with head and neck cancer and found that quitters had a significantly lower risk of a second primary neoplasm compared with those who continued to smoke (HR 0.34 (95%CI 0.24–0.56)) (Leon *et al*, 2009). In patients with superficial transitional cell carcinoma of the bladder, Fleshner *et al* reported data that allowed us to compare the risk of recurrence in patients who quit smoking after diagnosis to those who continued to smoke (Fleshner *et al*, 1999). Indirect comparison showed quitters were at lower risk of recurrence, but it was not significant (HR 0.71 (0.48–1.05)) (Aveyard *et al*, 2002). Taken together with our findings, the evidence appears inconclusive that quitting smoking lowers the risk of cancer progression in people with bladder cancer. However, overall there is stronger evidence that quitting improves cancer outcomes in UAT and lung cancer.

There are several potential mechanisms by which continued smoking may affect cancer-specific survival. Previous studies have shown that continued smoking may reduce the effectiveness of adjuvant cancer treatment (Dresler, 2003; Gritz *et al*, 2005; Zevallos *et al*, 2009; Chen *et al*, 2011; Gajdos *et al*, 2012). For example, tobacco smoke reduces plasma concentrations of chemotherapeutic agents as it is a potent inducer of the cytochrome P450 enzymes which metabolise several drugs in the liver, including some chemotherapies (Dresler, 2003; Petros *et al*, 2012). In addition to affecting treatment, constituents of tobacco smoke may also alter the behaviour of cancer cells or aid processes that support tumour development and progression (Yoshino and Maehara, 2007; Warren *et al*, 2014). Cigarette smoke contains many potential irritants and is a strong inflammatory stimulus (Lee *et al*, 2012; Warren *et al*, 2014), and evidence is been mounting that an inflammatory microenvironment aids development and progression of tumours (Balkwill and Mantovani, 2001; Aggarwal *et al*, 2006; Mantovani *et al*, 2008; Lee *et al*, 2012). It is possible that these mechanisms may be at play in the increased risk of death associated with continued smoking in lung and UAT cancers.

These results reinforce the need to provide active and ongoing smoking cessation support for people with cancer who smoke which is both a non-toxic and cost-saving intervention for the patient. In addition to reduced survival, previous studies have shown that continued smoking is associated with lower quality of life (Garces *et al*, 2014) increased pain (Daniel *et al*, 2009), treatment-related toxicity (Gritz *et al*, 2014; US Surgeon General, 2014b) and longer length of hospital stay in cancer patients (Erhunmwunsee and Onaitis, 2009). Many patients try to quit on diagnosis (Gritz *et al*, 1991), so those who relapse find themselves involuntarily trapped by their addiction. The lesson of the Lung Health Study, a study of people who continued smoking despite COPD, is that continued support for smoking cessation, despite early failure to quit, improves outcomes for patients (Scanlon *et al*, 2000). There is strong evidence that smoking cessation support in hospitalised patients that continues for at least a month after discharge increases rates of cessation (Rigotti *et al*, 2012). English (NICE) guidance recommends that patients accessing secondary care should be offered support to quit smoking within this setting, and for inpatients, this should include referral to intensive smoking cessation support which continues after discharge (Excellence. NICE, 2013). Despite this, support for smoking cessation is not well integrated into cancer care (Murray *et al*, 2012; Warren *et al*, 2013b) and future work should examine interventions to improve the health system to provide treatment for tobacco dependence.

## Conclusion

Most lung, UAT and bladder cancer patients who smoke at diagnosis continue smoking. However, quitting smoking reduces the risk of death in lung cancer and may do so in UAT cancer, but there is no evidence in this study that it does so in bladder cancer. The reduction in risk of death may be due to a decreased risk of cancer progression, but this needs further investigation.

## Figures and Tables

**Table 1:** Baseline characteristics of patients with lung, bladder and upper aero-digestive tract cancer by smoking exposure status

	**Lung cancer**			**Upper aero-digestive tract cancer**			**Bladder cancer**		
**Characteristics**	**Continued smoking (*****N=1129*****, 39%)**	**Quit smoking (*****N=784, 27%***)	**Status not updated (*****N=969, 34%***)	**Continued smoking (*****N=281, 37%***)	**Quit smoking (*****N=216, 29%***)	**Status not updated (*****N=260, 34%***)	**Continued smoking (*****N=850, 49%***)	**Quit smoking** (***N=356, 21%***)	**Status not updated** (***N=527, 30%***)
Gender (% male)	588 (52.1%)	364 (46.4%)	484 (49.9%)	197 (70.1%)	143 (66.2%)	181 (69.6%)	618 (72.7%)	269 (75.6%)	418 (79.3%)
Age (mean, s.d. years)	67.1 (9.97)	66.3 (9.04)	65.8 (10.4)	59.65 (10.86)	61 (10.83)	59.96 (11.2)	67.49 (10.7)	65.39 (10.8)	66.14 (11.5)
Patients with ONS linkage	625 (55.4%)	466 (59.4%)	544 (56.1%)	154 (54.8%)	120 (55.6%)	154 (59.2%)	494 (58.1%)	207 (58.15%)	312 (59.2%)
Alcohol status									
Non drinkers	264 (23.4%)	180 (22.9%)	216 (22.3%)	36 (12.8%)	35 (16.2%)	38 (14.6%)	228 (26.8%)	96 (27%)	126 (23.9%)
Ex-drinkers	103 (9.1%)	54 (6.9%)	49 (5%)	22 (7.8%)	13 (6%)	9 (3.5%)	85 (10%)	22 (6.2%)	20 (3.8%)
Light drinkers	101 (9%)	55 (7%)	75 (7.7%)	18 (6.4%)	25 (11.6%)	24 (9.2%)	65 (7.7%)	35 (9.8%)	55 (10.4%)
Moderate drinkers	100 (8.9%)	63 (8%)	65 (6.7%)	39 (13.9%)	29 (13.4%)	37 (14.2%)	79 (9.3%)	29 (8.2%)	38 (7.2%)
Heavy drinkers	30 (2.7%)	10 (1.3%)	20 (2.1%)	26 (9.3%)	11 (5.1%)	16 (6.2%)	16 (1.9%)	4 (1.1%)	8 (1.5%)
Drinkers (unknown amount)	60 (5.3%)	30 (3.8%)	25 (2.6%)	18 (6.4%)	8 (3.7%)	13 (5%)	28 (3.3%)	25 (7%)	8 (1.5%)
No record found	471 (41.7%)	392 (50%)	519 (53.6%)	122 (43.4%)	95 (43.8%)	123 (47.3%)	349 (41.1%)	145 (40.7%)	272 (51.6%)
IMD score^a^									
1	63 (5.6%)	68 (8.7%)	90 (9.3%)	9 (3.2%)	15 (6.9%)	19 (7.3%)	64 (7.5%)	36 (10.1%)	55 (10.4%)
2	105 (9.3%)	88 (11.2%)	104 (10.7%)	26 (9.3%)	20 (9.3%)	29 (11.2%)	98 (11.5%)	54 (15.2%)	72 (13.7%)
3	121 (10.7%)	76 (9.7%)	116 (12%)	31 (11%)	30 (13.9%)	29 (11.2%)	100 (11.8%)	45 (12.6%)	61 (11.6%)
4	149 (13.2%)	110 (14%)	124 (12.8%)	47 (16.7%)	26 (12%)	38 (14.6%)	120 (14.1%)	47 (13.2%)	77 (14.6%)
5	179 (15.9%)	124 (15.8%)	102 (10.5%)	41 (14.6%)	26 (12%)	34 (13.1%)	105 (12.4%)	23 (6.5%)	45 (8.5%)
Missing	512 (45.4%)	318 (40.6%)	433 (44.7%)	127 (45.2%)	99 (45.8%)	111 (42.7%)	363 (42.7%)	151 (42.4%)	217 (41.2%)
Comorbidities									
Asthma	127 (11.2%)	74 (9.4%)	56 (5.8%)	21 (7.5%)	11 (5.1%)	5 (1.9%)	67 (7.9%)	33 (9.3%)	15 (2.8%)
CKD	103 (9.1%)	62 (7.9%)	41 (4.2%)	12 (4.3%)	8 (3.7%)	9 (3.5%)	85 (10%)	24 (6.7%)	23 (4.4%)
COPD	370 (32.8%)	196 (25%)	143 (14.7%)	41 (14.6%)	27 (12.5%)	20 (7.6%)	140 (16.5%)	52 (14.6%)	25 (4.7%)
Diabetes	96 (8.5%)	85 (10.9%)	30 (3.1%)	20 (7.1%)	14 (6.5%)	5 (1.9%)	115 (13.5%)	34 (9.5%)	14 (2.7%)
Hypertension	263 (23.3%)	171 (21.8%)	130 (13.4%)	42 (14.9%)	61 (28.2%)	27 (10.4%)	223 (26.2%)	75 (21.1%)	78 (14.8%)
Peripheral arterial disease	103 (9.1%)	50 (6.4%)	40 (4.1%)	14 (5%)	10 (4.6%)	8 (3.1%)	62 (7.3%)	15 (4.2%)	19 (3.6%)
Stroke	102 (9%)	49 (6.2%)	41 (4.2%)	6 (2.1%)	10 (4.6%)	5 (1.9%)	57 (6.7%)	17 (4.8%)	19 (3.6%)
Psychosis	12 (1.1%)	6 (0.8%)	7 (0.7%)	2 (0.7%)	2 (0.9%)	1 (0.4%)	10 (1.2%)	2 (0.6%)	4 (0.8%)
Treatment									
Surgery	177 (15.7%)	264 (33.7%)	134 (13.8%)	60 (21.4%)	59 (27.3%)	68 (26.2%)	60 (7.1%)	44 (12.4%)	45 (8.5%)
Chemotherapy	338 (29.9%)	221 (28.2%)	309 (31.9%)	47 (16.7%)	33 (15.3%)	49 (18.8%)	113 (13.3%)	63 (17.7%)	76 (14.4%)
Radiotherapy	199 (17.6%)	98 (12.5%)	173 (17.9%)	75 (26.7%)	46 (21.3%)	63 (24.2%)	33 (3.9%)	7 (2.0%)	22 (4.2%)

Abbreviations: CKD=chronic kidney disease; COPD=chronic obstructive pulmonary disease; ONS=Office for National Statistics.

a IMD stands for index of multiple deprivation and is an area-based measure of socio-economic status which has been divided into quintiles, where 1 corresponds to least deprived and 5 to the most deprived.

**Table 2:** Risk of all-cause mortality, cancer-specific mortality and death due to index cancer in quitters compared with continuing smokers with lung, bladder and upper aero-digestive tract cancer

	**Ppts (*****n***)	**Total deaths (*****n***)	**All-cause mortality** **HR (95% CI)**	**Ppts (*****n***)	**Total deaths (*****n***)	**Cancer-specific mortality** **HR (95% CI)**	**Ppts (*****n***)	**Total deaths (*****n***)	**Death due to index cancer** **HR (95% CI)**
**Lung cancer**									
Unadjusted–missing exposure (extra category)	2881	2016	0.71 (0.63–0.79)	1635	1025	0.73 (0.62–0.85)	1635	954	0.72 (0.61–0.85)
Primary model (MI model–alcohol, SES, exposure)^a^	2881	2016	0.82 (0.74–0.92)	1635	1025	0.89 (0.76–1.04)	1635	954	0.90 (0.77–1.05)
Full case analysis with missing exposure as category^a^	835	617	0.82 (0.66–1.00)	835	529	0.87 (0.70–1.10)	835	496	0.87 (0.69–1.10)
Full case analysis (classify missing exposure as continued smokers)^a^	835	617	0.83 (0.69–1.00)	835	529	0.87 (0.70–1.10)	835	496	0.84 (0.68–1.04)
**Upper aero-digestive tract cancer**									
Unadjusted–missing exposure (extra category)	757	313	0.80 (0.60–1.08)	428	120	0.96 (0.61–1.51)	428	81	0.78 (0.45–1.37)
Primary model (MI model–alcohol, SES, exposure)^a^	757	313	0.81 (0.58–1.14)	428	120	0.84 (0.48–1.45)	428	81	0.75 (0.42–1.34)
Full case analysis with missing exposure as category^a^	233	106	0.72 (0.40–1.27)	233	71	0.88 (0.46–1.68)	233	49	0.71 (0.31–1.62)
Full case analysis (classify missing exposure as continued smokers)^a^	233	106	0.71 (0.41–1.20)	233	71	0.90 (0.43–1.63)	233	49	0.66 (0.31–1.42)
**Bladder cancer**									
Unadjusted–missing exposure (extra category)	1733	571	0.91 (0.73–1.14)	1013	213	1.23 (0.86–1.74)	1013	122	1.24 (0.77–1.99)
Primary model (MI model–alcohol, SES, exposure)^a^	1733	571	1.02 (0.81–1.30)	1013	213	1.23 (0.81–1.86)	1013	122	1.25 (0.71–2.20)
Full case analysis with missing exposure as category^a^	559	208	1.04 (0.72–1.52)	559	128	1.14 (0.71–1.83)	559	77	0.86 (0.44–1.65)
Full case analysis (classify missing exposure as continued smokers)^a^	559	208	1.00 (0.70–1.43)	559	128	1.02 (0.66–1.58)	559	77	0.76 (0.41–1.41)

Abbreviations: ppts=participants; (*n*)=number; MI=multiple imputation.

a Adjusted for age, gender, SES, co-morbidity, treatment, alcohol consumption.
